# Adsorption of Methyl Red on Poly(diallyldimethylammonium) Chloride-Modified Clay

**DOI:** 10.3390/ma18040766

**Published:** 2025-02-10

**Authors:** Simeng Li, Madjid Mohseni

**Affiliations:** Department of Chemical and Biological Engineering, University of British Columbia (UBC), 2360 East Mall, Vancouver, BC V6T 1Z3, Canada

**Keywords:** adsorption, dye contaminant, water treatment, modified clay composites, artificial neural network (ANN)

## Abstract

A novel, eco-friendly and cost-effective adsorbent, poly(diallyldimethylammonium) chloride (PDADMAC)-modified clay was developed to enhance its efficacy in removing Methyl Red (MR) from water. Different concentrations of PDADMAC solutions were evaluated during the synthesis and the effects of different operating conditions were investigated. The kinetic data closely followed the pseudo-first-order model, while equilibrium data were well described by Freundlich isotherm. MR removal efficiency decreased as solution pH or NaCl concentration increased, suggesting that electrostatic interaction plays a key role in the adsorption process. Regeneration studies using NaCl solutions revealed that a 1% NaCl solution effectively restored the adsorbent’s capacity. The findings indicate that PDADMAC clay is a promising and sustainable adsorbent for MR removal. Additionally, a three-layer backpropagation artificial neural network (ANN) was developed to predict the MR removal efficiency based on the initial MR concentration, pH, NaCl concentration, and adsorption time. Among these variables, pH was identified as the most influential factor. This approach provides valuable insight into the outcome prediction of a given adsorption process.

## 1. Introduction

Dyes have been widely used in various industries such as textile, leather, paper, rubber, printing and plastics [1]. More than 90,000 types of synthetic dyes are used commercially nowadays [2]. The textile industry consumes around 10,000 tons of dyes every year and releases around 100 tons of them into wastewaters. Dyes can affect aquatic life by reducing sunlight transmission through water and imparting toxicity [3]. They may also bring about significant health impacts to the population, such as dysfunctions of the kidney, reproductive system, liver, and central nervous system [4]. Methyl red (MR), an anionic azo dye, is one of the toxic, mutagenic, or carcinogenic dyes, and is commonly used in the textile and other industries [5,6,7].

Dyes are often resistant to biological degradation because of their complex structure and large molecular size [8]. Other physical or chemical treatment methods, such as advanced oxidation, electrocoagulation/electrodialysis, photo-catalysis, and reverse osmosis, have been studied for dye removal, but they are expensive and limited in their applicability [6]. Adsorption is a simple, effective, low-energy, and economically feasible technology to remove dyes from water, making it especially suitable for small plants with limited resources to implement advanced or expensive processes. Many researchers have studied the adsorption performance of various materials for MR removal. For instance, the Fe_3_O_4_@MIL-100 (Fe) adsorbent has demonstrated a high MR adsorption capacity, with the process identified as spontaneous and endothermic [9]. Similarly, Ag@Fe nanocomposites have shown effective MR adsorption, with equilibrium data fitting well to Langmuir, Freundlich, and Temkin isotherms [10]. UiO-66, another promising adsorbent, removes MR through multiple mechanisms, including electrostatic interactions, hydrogen bonding, π-π stacking, and the trapping of MR molecules within its porous structure [11]. Despite their effectiveness, these advanced adsorbents are relatively expensive and challenging to produce on a large scale, making them impractical for widespread dye wastewater treatment. In contrast, clays have gained attention as a more feasible adsorbent since they are eco-friendly, inexpensive, and found in high abundance in nature [12,13]. However, most clays, such as kaolinite, montmorillonite, and palygorskite, are ineffective for anionic dye removal through electrostatic interaction due to their negative surface charge [14]. To address this shortcoming, efforts have been made to modify the clay surface with various agents, such as surfactants, polymers, and amines [15,16,17,18]. For example, polycations can exchange with inner-layer cations of the clay, resulting in charge reversal on the clay surface and the formation of increased hydrophobic domains [19]. Poly(diallyldimethylammonium) chloride (PDADMAC) is a cationic and non-toxic polymer, and is widely used in water treatment, paper manufacturing, mining, and the biological field [20]. Although PDADMAC has been used for clay modification to improve its adsorption performance, most studies use powder-form clay composites [21,22,23]. Powder-form adsorbents generally have a large surface area leading to fast adsorption kinetics, but they are prone to agglomeration and cannot be directly used in fixed beds or any other flow-through systems because of the excessive pressure drops and the difficulty of separation from aqueous systems [24]. Thus, the use of granular composites is more feasible for large-scale application purposes.

This study aims to synthesize granular composites of PDADMAC clay and evaluate their MR adsorption performance under different conditions. To the best of the authors’ knowledge, this is the first open study to use granular composites of PDADMAC clay for MR adsorption. The composite adsorbents were evaluated for their physical characteristics as well as MR removal efficacy. The latter involved MR adsorption kinetics and isotherm studies, and the effects of solution pH and NaCl as a background solute on MR removal. Regeneration tests were also conducted to evaluate the reusability of the adsorbent. A feed-forward artificial neural network (ANN) with three layers (input layer, hidden layer, and output layer) was also developed to investigate the association between experimental variables (MR concentration, pH, NaCl concentration, and time) and outcome (MR removal efficiency) of the adsorption process.

## 2. Materials and Methods

### 2.1. Materials

Poly(diallyldimethylammonium) chloride (PDADMAC) (MW = 400–500 kDa), methyl red sodium salt, and sodium chloride were purchased from Sigma Aldrich (Oakville, ON, Canada). All chemicals were analytical grade or higher and were used without further purification. All solutions were prepared using deionized water (Milli-Q water with resistivity of 18.2 MΩ-cm). Clay (0.833–2 mm) was purchased from M.M BASICS (Victoria, BC, Canada), which was primarily marketed for use in soil mixes for succulents, cacti, and bonsai trees. It was rinsed with deionized water multiple times and dried in an oven overnight at 80 °C before use. The clays were in the form of irregularly shaped, granular particles with a coarse texture and a beige to light brown coloration. Under the adsorption test conditions of this study, the clay remained intact, and no visible fine particles were released into the solution.

### 2.2. PDADMAC Clay Synthesis

An amount of 1 g of clay was shaken with 100 mL of different concentrations of PDADMAC (0 *w*/*v* %, 2 *w*/*v* %, 4 *w*/*v* %, 8 *w*/*v* %, 10 *w*/*v* %, 12 *w*/*v* %, and 14 *w*/*v* %) solutions prepared in deionized water at 150 rpm for 4 h at room temperature. The clay was then washed with deionized water multiple times and dried at room temperature. To assess the desorption of PDADMAC, 50 mg of dried PDADMAC clay was then shaken with 100 mL of deionized water at 150 rpm for 24 h at room temperature. After separating the clay, the total organic carbon (TOC) of the water was verified to be less than 0.3 ppm to make sure that all the excess PDADMAC was washed off and the release of PDADMAC from clay was negligible.

### 2.3. Characterization and Analytical Methods

An FEI Quanta 650 scanning electron microscope (SEM) coupled with energy dispersive X-ray spectroscopy (EDX) (FEI Company, Hillsboro, OR, USA) was used to observe the micromorphology of the adsorbents and determine their elemental composition with an accelerating voltage of 20 kV. A Micromeritics Tristar II surface analyzer unit (Micromeritics Instrument Corporation, Norcross, GA, USA) was used to determine the textural characteristics of the adsorbents. The Brunauer–Emmett–Teller (BET) surface areas were determined from N_2_ isotherms in the range of 0.05 < P/P_o_ < 0.25, and the pore widths were determined using the Barrett–Joyner–Halenda (BJH) model. Thermogravimetric analysis (TGA) was conducted using Netzsch TG 209 F1 Libra^®^ (NETZSCH-Gerätebau GmbH, Selb, Bavaria, Germany) at a heating rate of 10 °C/min from 30 °C up to 1000 °C under a nitrogen atmosphere.

A UV–vis spectrophotometer (Cary 100, Agilent Technologies, Santa Clara, CA, USA) was used to determine MR concentration at 435 nm, at which MR has its maximum absorbance [11]. An ultraviolet (UV)/persulphate total organic carbon (TOC) analyzer (Sievers M5310C with autosampler, SUEZ Water Technologies & Solutions, Boulder, CO, USA) was used to determine the TOC concentration. An ion chromatograph (Dionex ICS-1100 with autosampler, Dionex Corporation, Sunnyvale, CA, USA) fitted with an AS22-Fast analytical column was used to detect chloride ions that were released during the adsorption process. An Oakton pH meter (Oakton Instruments, Vernon Hills, IL, USA) was used to measure solution pH.

### 2.4. Adsorption Experiments

To investigate the effect of the PDADMAC concentration used in the synthesis method, 100 mg PDADMAC clay synthesized with different PDADMAC concentrations was mixed with 100 mL of 10 ppm MR solution in a bench-top shaker at 150 rpm at room temperature for 22 h. The pH of the water was kept at around 6.5. Samples were taken at different time intervals and tested for their MR concentration. The PDADMAC clay with the best adsorption performance was used in further experiments.

The kinetics data of the PDADMAC clay with the best adsorption performance were fit into pseudo-first-order and pseudo-second-order equations as shown as below.

(1) $$\mathrm{Pseudo}\;\mathrm{first}\;\mathrm{order}:\;\frac{C}{C_{0}}=\left(1-\frac{C_{e}\;}{C_{0}}\;\right)exp^{-k_{1}t}+\frac{C_{e}\;}{C_{0}}$$

(2) $$\mathrm{Pseudo}\;\mathrm{second}\;\mathrm{order}:\;C=\frac{\left(C_{0}-C_{e}\right)M}{M+k_{2}t\left(C_{0}-C_{e}\right)}+C_{e}$$

where *C*_0_, *C*, and *C_e_* are the MR concentration at the initial time, at a specific time t, and at equilibrium, respectively (mg/L); *k*_1_ and *k*_2_ are the apparent rate constants of pseudo-first-order and pseudo-second-order adsorptions, respectively (min^−1^ and g media mg MR^−1^ min^−1^, respectively); *M* is the adsorbent concentration (g/L); and t is the contact time (min).

For the isotherm study, 50 mg of PDADMAC clay was exposed to 100 mL of MR solutions at concentrations ranging between 5 and 150 ppm in a shaker at 150 rpm at room temperature for 23 h. The pH of the water was kept around 6.5. The equilibrium data were fit into Freundlich isotherm and Langmuir isotherm equations as shown below.

(3) $$\mathrm{Freundlich}\;\mathrm{isotherm}:\;q_{e}=K_{f}{C_{e}}^{1/n}$$

(4) $$\mathrm{Langmuir}\;\mathrm{isotherm}:\;q_{e}=\frac{q_{m}bC_{e}}{1+bC_{e}}$$

where *q_e_* is the amount of MR adsorbed per unit mass of adsorbent at equilibrium (mg/g); *K_f_* is a Freundlich constant (mg/g) (L/mg)^1/n^; *n* is related to the energetic heterogeneity of the adsorbent surface; *q_m_* is the saturated maximum monolayer adsorption capacity per unit mass of media (mg/g); and *b* is a Langmuir affinity constant related to the binding energy of adsorption (L/mg). *q_e_* is calculated using the following equation:

(5) $$q_{e}=\frac{C_{0}-C_{e}}{M}$$

To investigate the effect of pH, 50 mg of PDADMAC clay was shaken with 100 mL 10 ppm MR solution at 150 rpm at room temperature. The pH of the solution was adjusted to 4, 5, 6.5, 8, and 9, respectively, using 0.1 M HCl or 0.1 M NaOH. To investigate the effect of NaCl, 50 mg of PDADMAC clay was shaken with 100 mL of solutions containing 10 ppm MR and 0–40 ppm NaCl at a pH of 6.5 at 150 rpm at room temperature. The equilibrium data were used for analysis.

All the adsorption tests were conducted in duplicate. The UV absorbance measurements were conducted in triplicate for each sample.

### 2.5. Regeneration Tests

An amount of 50 mg of PDADMAC clay was exposed to 100 mL of 50 ppm MR solution at a pH of 6.5 at 150 rpm at room temperature for 23 h. The used adsorbents were regenerated in 50 mL of 1%, 5%, or 10% NaCl solution in a shaker at 150 rpm at room temperature for 24 h. Then, they were washed with deionized water multiple times. The regenerated adsorbents were reused in the next cycle of adsorption experiments. The adsorption–regeneration tests were continued for 6 cycles in total. A control test was also conducted by regenerating used PDADMAC clay in deionized water.

### 2.6. ANN Development

Machine learning is an emerging field of artificial intelligence that can learn from the previous data, generate models, and predict the outcome of a certain process [25]. Among them, the artificial neural network (ANN) is well known for having a large number of processing units called neurons that can reproduce complex and non-linear relationships [26]. It has many advantages over conventional modeling, such as the ability to handle noisy data from dynamic and non-linear systems, especially when the underlying physical relationships are difficult to fully understand [27]. In this study, a feed-forward ANN with three layers (input layer, hidden layer, and output layer) was built in MATLAB (R2023b) to forecast the removal efficiency of MR for a given initial MR concentration, pH, NaCl concentration, and adsorption time. Sigmoid and linear functions were employed at the hidden layer and output layer, respectively. The experimental datasets were randomly subdivided into three categories: 70% as the training dataset, 15% as the validation dataset, and 15% as the testing dataset. The range of input data was 5–40 ppm of MR, 0–40 ppm of NaCl, 4–9 as pH, and 90–1380 min as adsorption time. The input data were normalized before being fed into the network using the following equation:

(6) $$y=0.8\times \left(\frac{x_{i}-x_{min}}{x_{max}-x_{min}}\;\right)+0.1$$

where *y* is the normalized value of input data *x_i_*; and *x_max_* and *x_min_* are the maximum and minimum values of *x_i_*, respectively.

Three different algorithms (Levenberg–Marquardt, Bayesian regularization, and Scaled conjugate gradient backpropagation) with four neurons in the input layer, ten neurons in the hidden layer, and one neuron in the output layer were tried in order to determine the best algorithm for modifying the weights of the neural network. The modeling performance was evaluated based on mean squared error (MSE) and determination of coefficient (R^2^). After determining the best algorithm, different numbers of neurons in the hidden layer were tried to determine the best ANN model. A sensitivity analysis was further conducted to examine the relative significance of the experimental variables using weights of the best generated neural network [28].

(7) $$I_{j}=\frac{\sum_{m=1}^{N_{h}}((\left|IW_{jm}\right|/\;\sum_{k=1}^{N_{i}}\left|IW_{km}\right|)\times |LW_{mn}|\;)}{\sum_{k=1}^{N_{i}}(\sum_{m=1}^{N_{h}}((\left|IW_{km}\right|/\;\sum_{k=1}^{N_{i}}\left|IW_{km}\right|)\times |LW_{mn}|\;))}$$

where *I_j_* is the proportional weight of the variable *j*; *N_h_* and *N_i_* are the number of neurons in the hidden layer and input layer, respectively; *IW* is the weight between the input and hidden layers; *LW* is the weight between the hidden and output layers; *k* is input neuron; *m* is the hidden neuron; and *n* is the output neuron.

## 3. Results

### 3.1. Effect of PDADMAC Concentration

**Figure 1:**
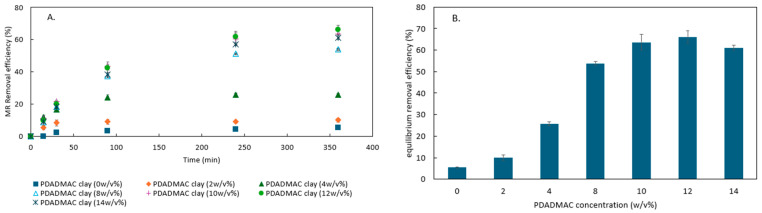
(**A**) The methyl red (MR) removal efficiency of poly(diallyldimethylammonium) chloride (PDADMAC) clay synthesized with different PDADMAC concentrations (PDADMAC concentrations in water at the beginning of synthesis procedure). The MR concentration was not further changed after 6 h of adsorption. (**B**) The equilibrium MR removal efficiency of PDADMAC clay synthesized with different PDADMAC concentrations.

To determine the effect of PDADMAC concentration on the adsorption performance, PDADMAC clay synthesized with different PDADMAC concentrations was mixed with a 10 ppm MR solution for 6 h, and the results are shown in Figure 1. The raw clay (PDADMAC clay (0 *w*/*v* %)) showed negligible MR adsorption capacity. As the polymer concentration increased to 12 *w*/*v* %, the MR adsorption performance increased, indicating that the increase in polymer loading leads to more positive binding sites on the adsorbent for MR adsorption. However, the adsorption of MR decreased when PDADMAC further increased to 14 *w*/*v* %. It is hypothesized that the presence of an excess of long-chain polymer blocked some pores, thereby decreasing the available surface area for adsorption. This hypothesis was evaluated through BET analysis, presented in the next section. A similar trend was also reported by other studies involving PDADMAC clay for the herbicide imazapyr and humic acid adsorption [23,29]. However, those studies used powder-form clay and the decrease in contaminant adsorption at high PDADMAC loadings was associated with clay aggregation caused by contaminant bridging which decreased accessible surface area. Since the PDADMAC clay synthesized with 12 *w*/*v* % polymer showed the best adsorption performance, it was used in further characterization, adsorption, and regeneration experiments.

### 3.2. Characterization of Adsorbent

**Figure 2:**
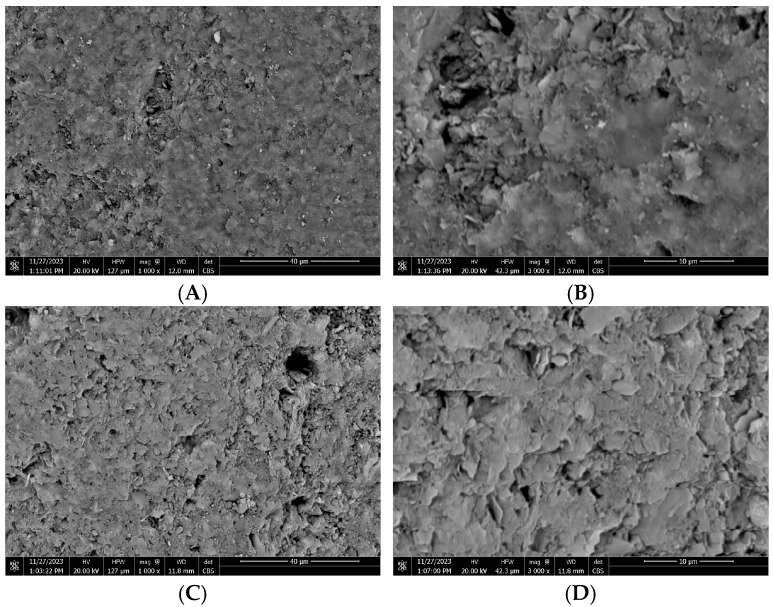
SEM images of (**A**,**B**) raw clay and of (**C**,**D**) PDADMAC clay synthesized with 12 *w*/*v* % polymer.

The SEM images of raw clay and PDADMAC clay are shown in Figure 2. The SEM image of raw clay reveals a relatively rough and irregular surface morphology with compact, low-porosity regions, indicating that its surface area is likely to be low. No significant change in morphology is observed after loading the clay with PDADMAC. Thus, only a thin layer of polymer is coated on the clay surface, confirming that it would not alter the original structure of clay. The EDX result shows that raw clay has O, Si, Al, Mg, Fe, K, Ca, Ti, C, and N (shown in Figure S1 in Supplementary Materials), suggesting the presence of typical clay minerals such as SiO_2_, Al_2_O_3_, Mg(OH)_2_/MgCO_3_, Fe_2_O_3_, TiO_2_, KOH/K_2_CO_3_, and Ca(OH)_2_/CaCO_3_. The PDADMAC clay has Cl in addition to the elements of raw clay since PDADMAC contains Cl. The distribution of Cl on PDADMAC clay is uniform (shown in Figure S2 in Supplementary Materials), suggesting that the potential ion exchange sites are distributed on the clay surface evenly.

**Figure 3:**
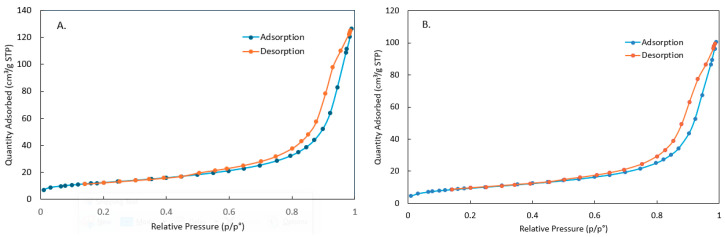
N_2_ isotherms of (**A**) raw clay and (**B**) PDADMAC clay synthesized with 12 *w*/*v* % polymer.

The N_2_ isotherms of raw clay and PDADMAC clay are shown in Figure 3. The isotherms are classified as type II and the hysteresis loops are H3. The H3 loops may indicate that the adsorbent consists of macropores which are not completely filled by N_2_ condensate [30]. The isotherms do not show a steep initial portion at low relative pressure, which may indicate that the proportion of micropores (<2 nm) is low [31]. The BET surface area of raw clay and PDADMAC clay are 44.32 m^2^/g and 34.92 m^2^/g, respectively. The average pore width calculated by the BJH method of raw clay and PDADMAC clay are 18.79 nm and 18.97 nm, respectively. Thus, PDADMAC loading decreases the surface area of the clay, but does not affect the average pore width. This is likely due to some pores being blocked by the PDADMAC, leading to the inaccessibility of the internal surface to nitrogen molecules. The reduction in clay surface area after loading with PDADMAC or other polymers has also been reported in other studies [17,21,32,33]. Since the adsorption of MR molecules relies on the available surface area, the decrease in surface area explains the reduced MR removal efficiency at very high polymer concentrations as shown in Figure 1. Although PDADMAC clay synthesized with 12 *w*/*v* % polymer has a smaller BET surface area than raw clay, the introduction of new adsorption sites by the polymer more than compensates for the reduction in surface area, leading to an enhanced removal performance.

The TGA curves of clay, PDADMAC clay, and PDADMAC are shown in Figure S3 in Supplementary Materials. The weight loss that appeared below 200 °C was likely to be attributed to the elimination of adsorbed water. PDADMAC exhibited a rapid mass loss between 300 °C and 500 °C due to the polymer’s decomposition, while the major mass loss of PDADMAC clay occurred between 600 °C and 700 °C. Thus, after combining with clay, the thermal stability of PDADMAC clay was improved compared to pure PDADMAC. Raw clay only exhibited a 2.68% mass loss due to clay dehydroxylation, while PDADMAC clay exhibited a 10.31% mass loss due to the additional decomposition of PDADMAC. Thus, PDADMAC clay was approximated to contain 7.63 mass% of PDADMAC. The Fourier-transform infrared spectroscopy (FTIR) results are shown in Figure S4 in Supplementary Materials.

### 3.3. Adsorption Results

#### 3.3.1. Adsorption Kinetics and Isotherm

**Figure 4:**
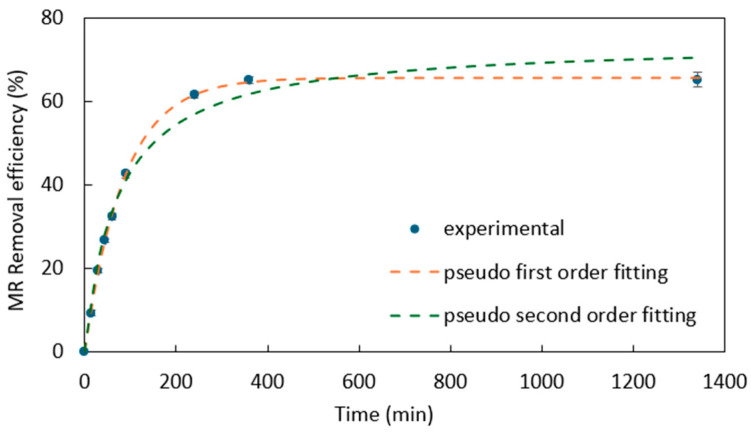
Experimental and pseudo-first-/second-order-fitted MR adsorption kinetics of PDADMAC clay. MR initial concentration: 10 ppm; adsorbent dose: 1 g adsorbent/L of water.

The initial uptake rate of MR is rapid, and then it gradually slows down until reaching an equilibrium state. Pseudo-first-order and pseudo-second order equations have been used to fit the kinetics of MR adsorption in many studies [6,7,34]. The results of this study are shown in Figure 4 and Table 1. The regression correlation coefficient (R^2^) of pseudo-first-order equation is a bit higher than that of pseudo-second-order equation, and pseudo-first-order equation leads to a fitted equilibrium concentration closer to the actual value (3.4 ± 0.2 mg/L in this test). Thus, pseudo-first-order equation could describe the adsorption kinetics better than pseudo-second-order, which implies that chemisorption is not the main mechanism of this adsorption process and one MR molecule is adsorbed onto one active site of adsorbent [6,35]. The pictures of the kinetics test, UV absorbance curve, and the appearance of different concentrations of MR solutions are shown in Figures S5–S7. 

**Figure 5:**
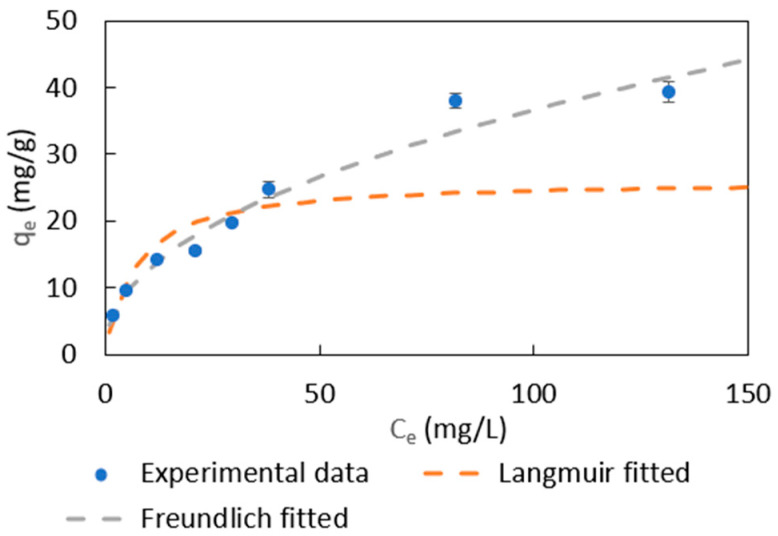
Comparison of Freundlich and Langmuir isotherms fittings for MR adsorption on PDADMAC clay.

The equilibrium data of 50 mg of PDADMAC clay in 100 mL of 5–150 ppm MR solution were fitted to Freundlich and Langmuir isotherms as shown in Figure 5 and Table 2. Based on the R^2^ value and root mean square error (RMSE) value, the Freundlich isotherm could better describe the MR adsorption equilibrium. The Langmuir isotherm fails to describe the adsorption behavior at high MR concentrations. It indicates that different interaction types are involved in the adsorption process, which meets the conclusion in Section 3.3.3. The value of *n* is greater than 1, indicating a strong interaction between MR and PDADMAC clay.

#### 3.3.2. Effect of pH and Inorganic Compound

**Figure 6:**
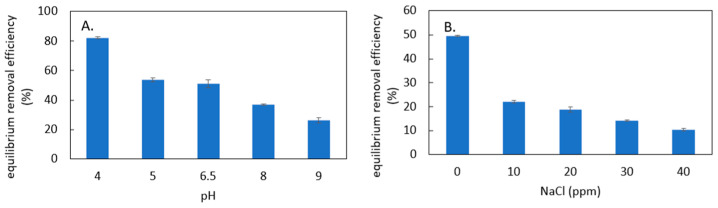
Equilibrium MR removal efficiency in solutions with (**A**) different pH and (**B**) different NaCl concentrations.

The effect of the solution’s pH on MR adsorption performance is shown in Figure 6A. It shows that the MR removal efficiency gradually decreases as pH increases. This trend agrees with the results reported in other MR adsorption studies using different adsorbents [5,9]. The reason for this observation is that adsorbent’s surface would become less positively charged as pH increases, and if the solution’s pH is above the point of zero charge of the adsorbent, the surface charge of the adsorbent would be negative. Thus, the electrostatic attraction between the adsorbent and the MR would be lower at a high pH. In addition, there is more competition between hydroxyl ions and MR for the adsorption sites as pH increases, which also leads to a reduction in MR adsorption capacity at a high pH.

The effect of NaCl concentration in solution on MR adsorption performance is shown in Figure 6B. NaCl is considered as a key solute present in water contaminated with MR since it is a very common chemical used in dyeing and printing processes in industry to enhance the fixation between dyes and fabrics [36,37]. Figure 6B shows that the MR removal efficiency decreases significantly as NaCl concentration increases. This trend agrees with the results reported in other anionic dye adsorption studies using different adsorbents [11,38]. The likely explanation for this result is the presence of greater competition between chloride and MR for binding sites as the NaCl concentration increases. In addition, as ionic strength increases, the screening effect would reduce the thickness of the electric double layer hence reducing the electrostatic attraction between the MR and the positively charged adsorption sites.

These findings suggest that PDADMAC clay is more effective in low-pH conditions and in water with a low salinity. However, adjusting the pH or decreasing the ionic content of wastewater may increase operational costs in practical applications. This highlights the need for a balanced approach that considers both the treatment efficacy and cost-effectiveness. Further research could explore alternative methods for improving MR removal in water with high pH or NaCl concentrations, such as pre-treatment steps, optimizing adsorbent design, or incorporating complementary treatment technologies.

#### 3.3.3. Adsorption Mechanism

**Figure 7:**
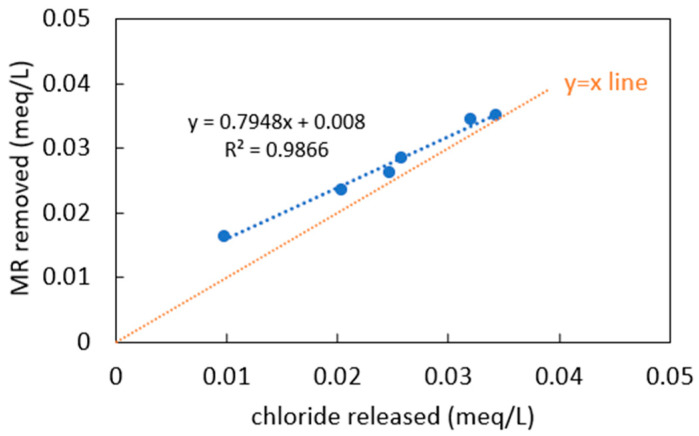
Relationship between amount of MR removed and amount of chloride released.

Chloride ions were observed to be released into the solution when the MR was adsorbed to the PDADMAC clay. As shown in Figure 7, the amount of MR removed and the amount of chloride released follow a positive linear relationship. Compared to the 45° line, the amount of MR removed is slightly larger than the amount of chloride released. Thus, the adsorption of MR on PDADMAC clay is mainly contributed to by the equivalent exchange of chloride ions with MR molecules, with a small contribution from other mechanisms such as hydrophobic interaction. This agrees with the results of the pH and inorganic compound effects study which implies that electrostatic interaction is the main adsorption mechanism of PDADMAC clay. It also suggests that a brine solution should be able to regenerate the used clay to recover most of its adsorption capacity. This is promising since it avoids harsh conditions or environments (such as high temperature, low/high pH) for regeneration which present major challenges to industry and contribute significant safety and environmental concerns. A schematic representation of the interaction mechanism is shown in Figure S8 in Supplementary Materials.

### 3.4. Regeneration of Adsorbents

**Figure 8:**
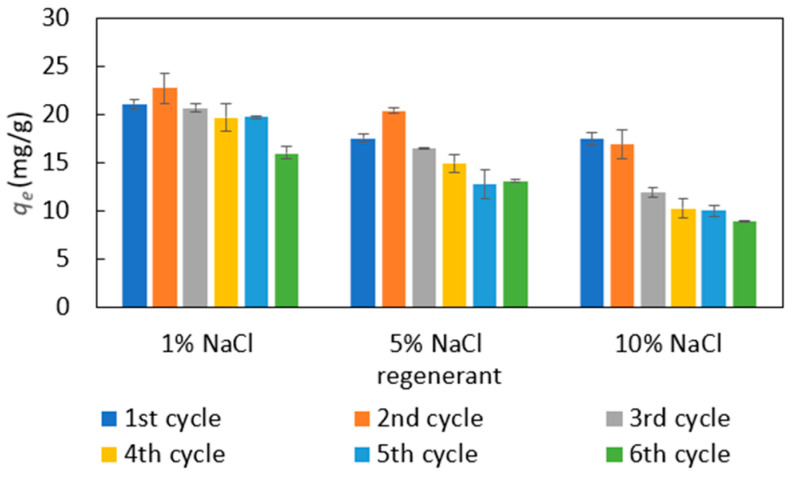
Regeneration tests of PDADMAC clay using different concentrations of NaCl solutions for 6 cycles. First cycle used new adsorbents; later cycles used regenerated adsorbents.

A good regeneration capability for adsorbents is critical to reduce the environmental impact of the used adsorbents and to improve their economic competitiveness. To determine the reusability of PDADMAC clay, the used clays were regenerated with different concentrations of NaCl solutions between adsorption cycles, and their adsorption efficacies were investigated as shown in Figure 8. NaCl is used as regenerant since it is inexpensive and highly soluble, and has been widely used to regenerate ion-exchange resins [39,40]. As shown in Figure 8, 1% NaCl could efficiently regenerate PDADMAC clay and recover its adsorption capacity. It also proves that MR is adsorbed on PDADMAC clay mostly through electrostatic interaction. The regeneration performance decreases when the NaCl concentration increases to 10%, which may be due to the low solubility of MR in water at a high salt concentration, known as the ‘salting-out’ effect. In highly saline environments, water molecules preferentially hydrate salt ions, leaving fewer water molecules available to solvate MR molecules. This reduced solubility of MR molecules may hinder their desorption from the adsorbent during the regeneration process. The higher regeneration efficiency of a dilute salt solution has also been reported in other studies where anionic contaminants are removed by the ion exchange mechanism [41]. The control test shows that deionized water has a negligible regeneration effect where the removal efficiency decreases sharply after the first adsorption cycle (as shown in Figure S9 in Supplementary Materials).

### 3.5. ANN Results

To investigate the association between experimental variables and MR removal efficiency, three different algorithms with four neurons in the input layer, ten neurons in the hidden layer, and one neuron in the output layer were tried as shown in Table 3.

The Levenberg–Marquardt backpropagation algorithm seems to have the best modeling performance and fastest development rate among these three algorithms. Thus, it was selected as the training algorithm for further investigation.

With four neurons in the input layer and one neuron in the output layer, 1–20 neurons in the hidden layer were tried in the ANN model built with the Levenberg–Marquardt algorithm. As shown in Figure S10 in Supplementary Materials, 11 neurons in the hidden layer seem to lead to the best performance with R^2^ = 0.990 for the training data, R^2^ = 0.981 for the validation data, and R^2^ = 0.981 for the test data. The scatter plots of predicted versus actual values for training, validation, testing, and all data are shown in Figure S11. All of the correlation coefficients are close to 1 and almost all data disperse around the 45° line, thus the developed 4-11-1 ANN structure could predict MR removal efficiency accurately for a given initial MR concentration, pH, NaCl concentration, and adsorption time. The weights of the best developed ANN are shown in Table 4. It is important to note that the developed ANN was trained and tested using data within specific ranges of these parameters. If the water characteristics or flow conditions differ significantly from those used in the study, the model’s applicability may need to be reassessed, and the network may need to be retrained or restructured accordingly to ensure accurate predictions under new conditions.

A two-stage sensitivity analysis was conducted to examine the relative importance of the experimental variables. The first-stage analysis uses the weights of the best generated neural network listed in Table 4. As shown in Table 5, all variables have a strong effect on adsorption performance. pH seems to be the most influential variable for MR adsorption within the tested range, followed by initial MR concentration and NaCl concentration.

The second-stage analysis was conducted using different combinations of input variables. The performance of grouping one, two, three, and four input parameters are examined using the ANN model with a Levenberg–Marquardt function and 11 hidden neurons. The input variables are V_1_ (initial MR concentration), V_2_ (pH), V_3_ (initial NaCl concentration), and V_4_ (adsorption time). As shown in Table 6, MSE values generally decrease as the number of input variables used in the ANN model increases. V_2_ shows the minimum MSE of 0.0144 when a single variable is used as the input. The minimum MSE decreases up to 0.0063 when two input variables, V_2_ + V_3_, are used. It further decreases up to 0.0041 when three input variables, V_1_ + V_2_ + V_3_, are used. Four input variables, V_1_ + V_2_ + V_3_ + V_4_, further decreases the MSE to 0.0010. This analysis further proves that V_2_ (pH) is the most influential parameter, and all four parameters have a strong effect on the MR removal efficiency.

## 4. Conclusions

In this study, PDADMAC clay granular composites were synthesized and evaluated for their MR adsorption performance under various conditions. PDADMAC clay synthesized with 12 *w*/*v* % PDADMAC solution showed the best adsorption performance. The surface analysis showed that PDADMAC loading decreased surface area of the clay, but did not change the average pore width. The kinetics data were fit into pseudo-first-order and pseudo-second-order equations, and the Freundlich isotherm effectively described the equilibrium behavior. The MR removal efficiency decreased as solution pH or NaCl concentration increased, suggesting that electrostatic interaction plays a significant role in the adsorption process. A 1% NaCl solution could efficiently regenerate PDADMAC clay, indicating the good reusability of this adsorbent. These findings indicate that PDADMAC clay is a promising, low-cost adsorbent for MR removal. The optimized 4-11-1 ANN structure, trained using the Levenberg–Marquardt backpropagation algorithm, accurately predicted MR removal efficiency based on initial MR concentration, pH, NaCl concentration, and adsorption time. The implications of this study are significant for both pilot-scale and industrial-scale water treatment applications. The ability to regenerate the adsorbent with minimal loss in performance ensures cost-effectiveness and sustainability, while the ANN model offers a powerful tool for performance prediction and adsorption process optimization. This study also provides a comprehensive foundation for future research on modified clay composites and their application in water treatment systems, addressing both economic feasibility and environmental sustainability.

## Figures and Tables

**Table 1:** Fitted adsorption rates of MR adsorption on PDADMAC clay.

Pseudo First Order			Pseudo Second Order		
*k*_1_ (min^−1^)	*C_e_* (mg/L)	R^2^	*k*_2_ (g /mg min)	*C_e_* (mg/L)	R^2^
0.0116 ± 0.0001	3.4 ± 0.1	0.999	0.00187 ± 0.00003	2.5 ± 0.1	0.981

**Table 2:** Fitted Freundlich and Langmuir isotherm parameters for MR adsorption.

Isotherm	Parameters	Values
Freundlich	*K_f_* (mg/g)(L/mg)^1/n^	4.4 ± 0.5
	*n*	2.2 ± 0.2
	R^2^	0.981
	RMSE	3.68
Langmuir	*q_m_* (mg/g)	26.2 ± 5.5
	*b* (L/mg)	0.15 ± 0.05
	R^2^	0.935
	RMSE	8.07

**Table 3:** Three different algorithms applied for artificial neural network (ANN) development.

	Epoch	MSE	R^2^
Levenberg–Marquardt	12	0.00131	0.982
Bayesian regularization	508	0.00272	0.973
Scaled conjugate gradient backpropagation	15	0.01604	0.745

**Table 4:** The weights of the best developed ANN.

Neuron	*IW*				*LW*
	MR Concentration	pH	NaCl Concentration	Time	MR Removal Efficiency
1	0.473	−1.883	−0.665	−0.123	0.200
2	1.298	−1.925	−0.937	1.287	−0.030
3	0.971	1.783	1.380	−0.239	0.341
4	0.591	1.471	−2.729	0.118	0.337
5	1.815	2.027	0.125	−0.171	−0.371
6	−1.393	3.113	−0.273	−0.490	−0.578
7	0.965	0.278	1.025	2.436	−0.268
8	−0.384	0.926	−1.995	0.067	−0.117
9	−0.599	−0.500	0.209	−2.057	−0.336
10	1.515	1.645	0.849	−0.291	−0.480
11	−0.635	1.453	−0.923	1.894	0.218

**Table 5:** Relative significance of input variables.

Input Variable	Relative Significance (%)
MR concentration	24.25
pH	37.83
NaCl concentration	20.39
Time	17.53

**Table 6:** Evaluation of possible combinations of input variables.

Combination	Epoch	MSE	R^2^
V_1_	3	0.0323	0.316
V_2_	3	0.0144	0.777
V_3_	3	0.0302	0.430
V_4_	5	0.0384	0.200
V_1_ + V_2_	6	0.0094	0.860
V_1_ + V_3_	5	0.0235	0.585
V_1_ + V_4_	8	0.0315	0.376
V_2_ + V_3_	5	0.0063	0.908
V_2_ + V_4_	8	0.0127	0.805
V_3_ + V_4_	12	0.0264	0.513
V_1_ + V_2_ + V_3_	7	0.0041	0.942
V_1_ + V_2_ + V_4_	9	0.0140	0.800
V_1_ + V_3_ + V_4_	11	0.0220	0.626
V_2_ + V_3_ + V_4_	12	0.0051	0.928
V_1_ + V_2_ + V_3_ + V_4_	5	0.0010	0.986

## Data Availability

The original contributions presented in the study are included in the article/Supplementary Materials; further inquiries can be directed to the corresponding author.

## Supplementary Materials

The following supporting information can be downloaded at: https://www.mdpi.com/article/10.3390/ma18040766/s1, Figure S1: Distribution of elements of raw clay; Figure S2: Distribution of elements of PDADMAC clay synthesized with 12 *w*/*v* % polymer; Figure S3: Thermogravimetric analysis of clay, PDADMAC clay, and PDADMAC; Figure S4: FTIR spectra of clay powder and PDADMAC clay powder; Figure S5: Pictures of the PDADMAC clay MR adsorption kinetics test. MR initial concentration: 10 ppm; Adsorbent dose: 1 g adsorbent/L water; Figure S6: UV absorbance at 435 nm of PDADMAC clay MR adsorption kinetics test. MR initial concentration: 10 ppm; Adsorbent dose: 1 g adsorbent/L water; Figure S7: Pictures of different concentrations of MR solutions; Figure S8: Interaction mechanisms between ionized MR and PDADMAC in water; Figure S9: Regeneration tests of PDADMAC clay using deionized water for 5 cycles; Figure S10: The MSE of the ANN with a different number of neurons in the hidden layer; Figure S11: Scatter plots of predicted versus actual values for training, validation, testing, and all data. Reference [42] is cited in the supplementary materials.
