# Supplementary material for: Adsorption of Methyl Red on Poly(diallyldimethylammonium) Chloride-Modified Clay
Source: Materials (Basel). 2025 Feb 10;18(4):766. doi: 10.3390/ma18040766 (PMC11857668; doi:10.3390/ma18040766)
Supplement: Supplementary file 1 [file materials-18-00766-s001.zip › materials-3360615-supplementary.pdf]

Adsorption of Methyl Red on Poly(diallyldimethylammonium) Chloride-Modified Clay

Simeng Li and Madjid Mohseni *

Department of Chemical and Biological Engineering, University of British Columbia (UBC), 2360 East Mall, Vancouver, BC V6T 1Z3, Canada; simengli@student.ubc.ca
* Correspondence: madjid.mohseni@ubc.ca

Supplementary Materials

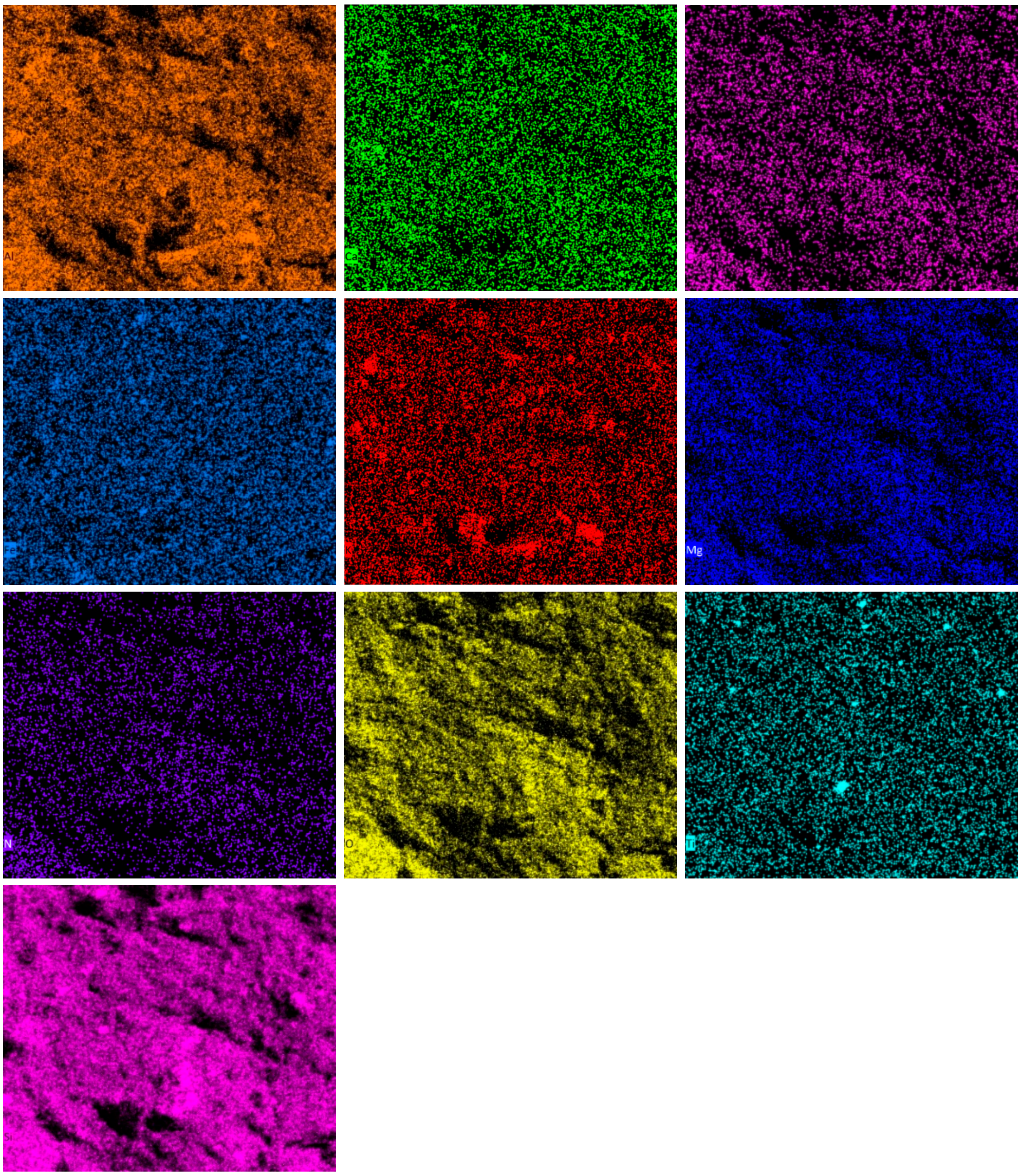

Figure S1. Distribution of elements of raw clay.

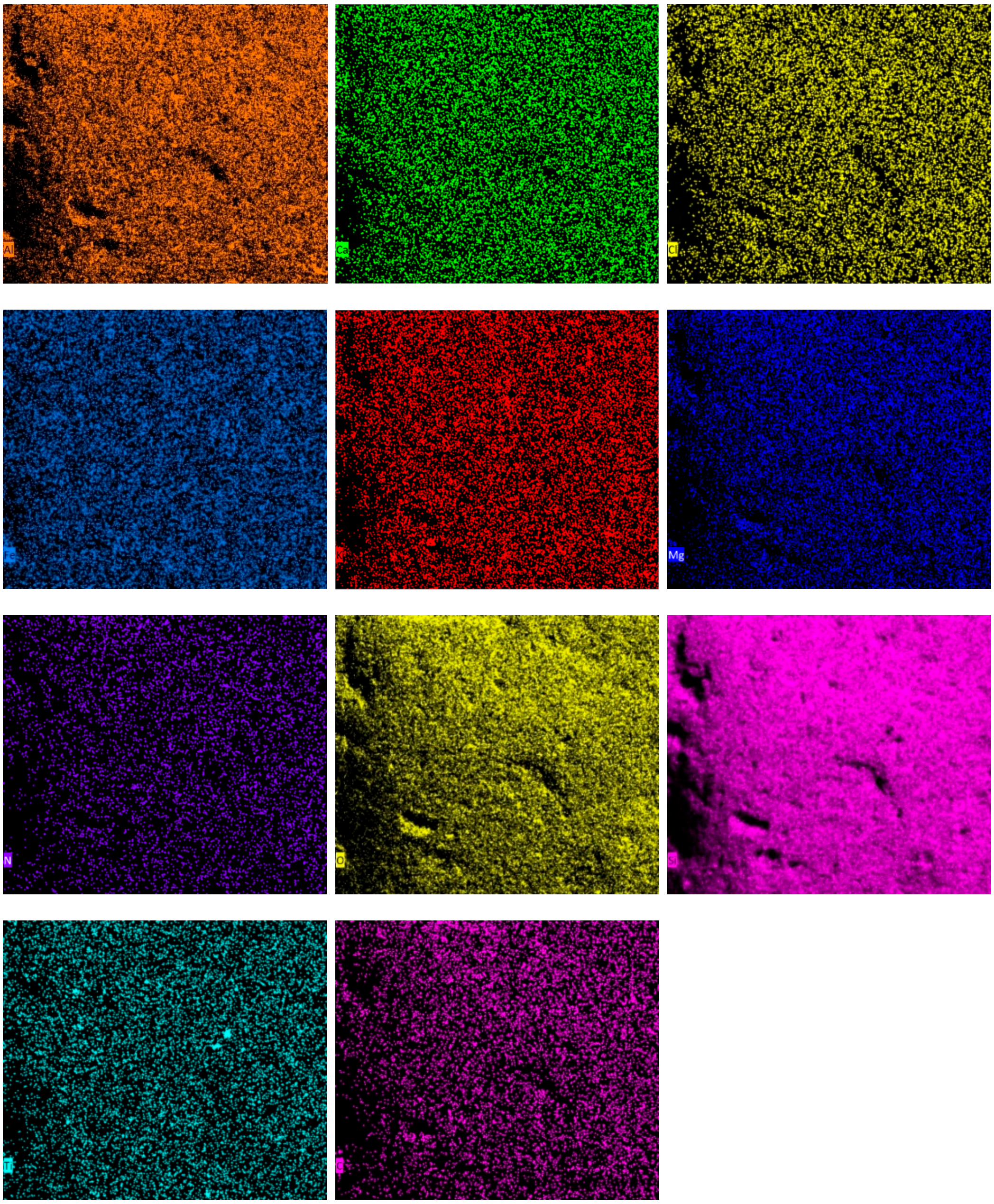

Figure S2. Distribution of elements of PDADMAC clay synthesized with 12w/v% polymer.

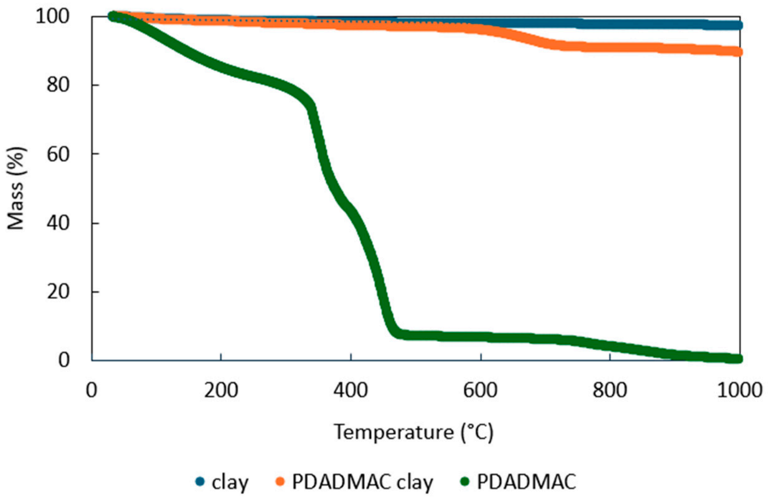

Figure S3. Thermogravimetric analysis of clay, PDADMAC clay and PDADMAC.

Fourier-transform infrared spectroscopy (FTIR) of clay and PDADMAC clay were measured using a Nicolet™ iS50 FTIR Spectrometer. The materials were ground into powders before measurement to minimize background interference.

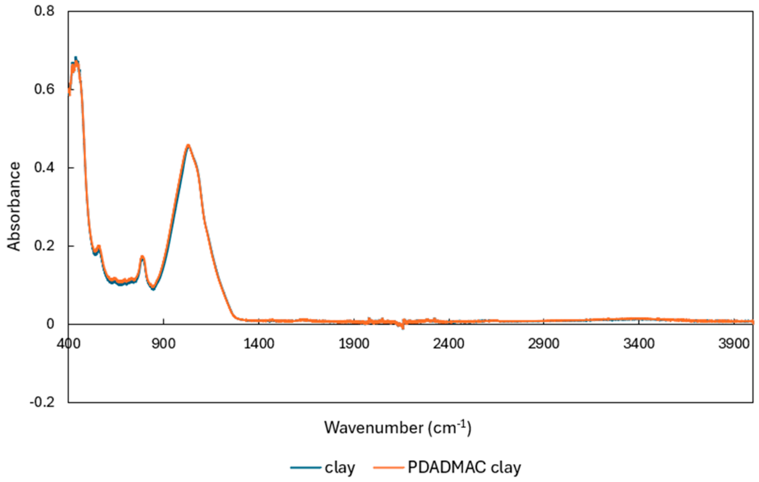

Figure S4. FTIR spectra of clay powder and PDADMAC clay powder.

The peak at 1030 cm^{-1} corresponds to Si–O stretching vibration in the clay [1]. However, the FTIR spectrum of PDADMAC clay powder is not significantly different from that of clay powder. This is likely because the PDADMAC modification is majorly on the surface of clay. Grinding the clay into powder for analysis exposes its internal structure, which does not reflect its surface modification effectively.

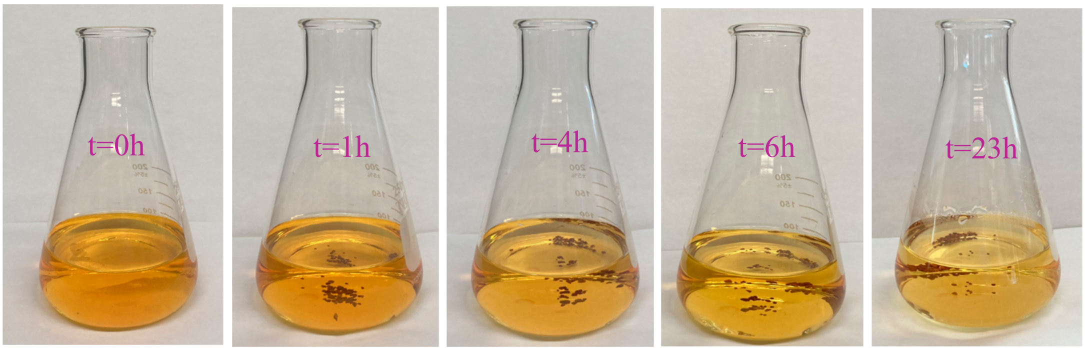

Figure S5. Pictures of PDADMAC clay MR adsorption kinetics test. MR initial concentration: 10ppm; Adsorbent dose: 1g adsorbent /L water.

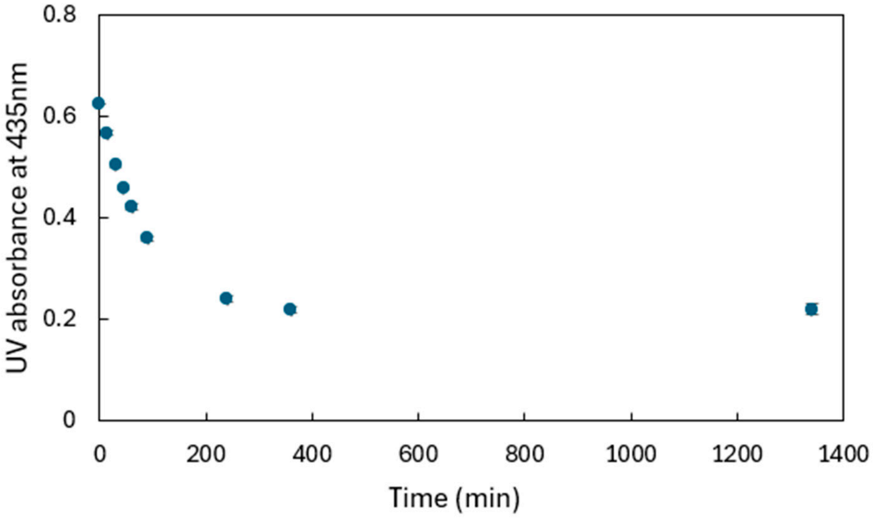

Figure S6. UV absorbance at 435nm of PDADMAC clay MR adsorption kinetics test. MR initial concentration: 10ppm; Adsorbent dose: 1g adsorbent /L water.

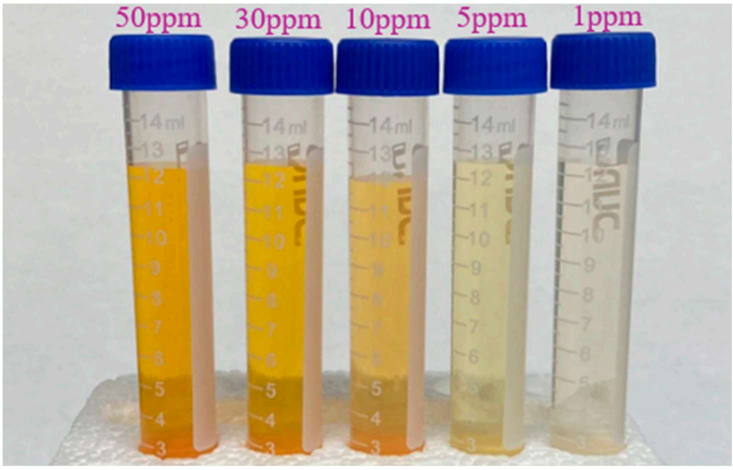

Figure S7. Pictures of different concentrations of MR solutions.

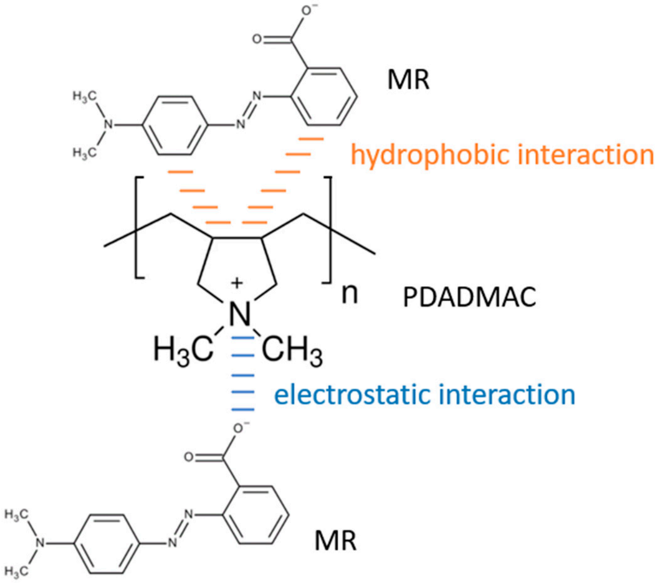

Figure S8. Interaction mechanisms between ionized MR and PDADMAC in water.

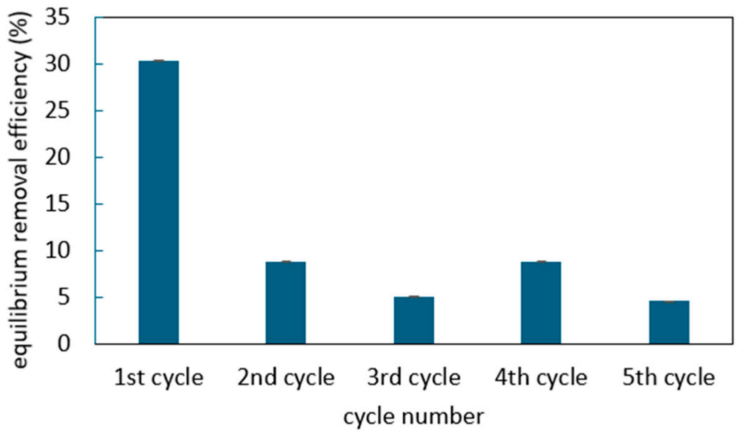

Figure S9. Regeneration tests of PDADMAC clay using deionized water for 5 cycles. 1st cycle used new adsorbents; later cycles used regenerated adsorbents. The error bar is calculated from standard deviation of the UV absorbance readings.

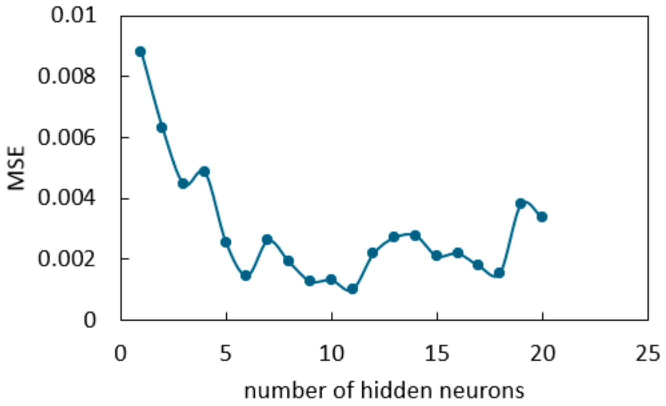

Figure S10. MSE of ANN with different number of neurons in the hidden layer.

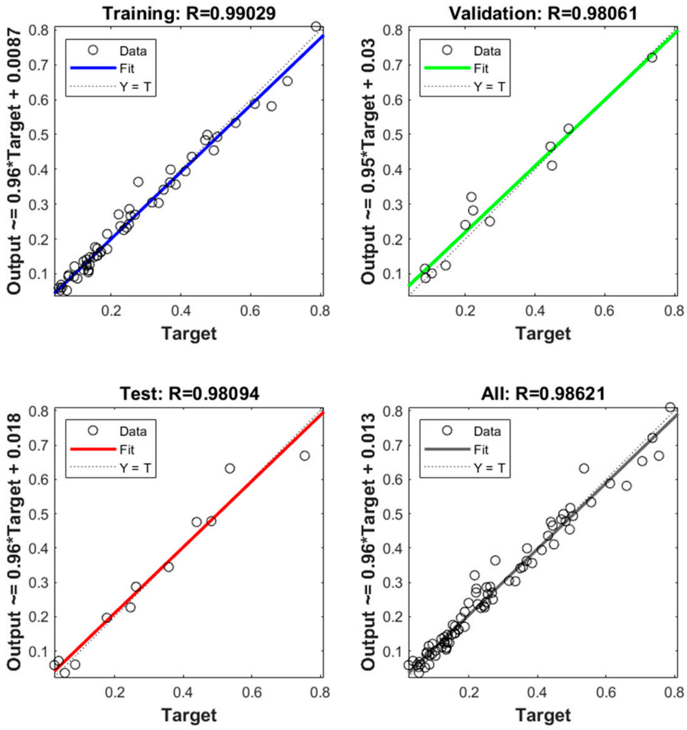

Figure S11. Scatter plots of predicted versus actual values for training, validation, testing and all data.

References

1. Madejová, J. FTIR Techniques in Clay Mineral Studies. *Vib. Spectrosc.* **2003**, *31*, 1–10, doi:10.1016/S0924-2031(02)00065-6.
